# Association of Hypothyroidism and Anti-Thyroid Antibodies With Preterm Delivery: A Cross Sectional Study 

**Published:** 2017-12

**Authors:** Tahereh Behroozi-Lak, Ameneh Akbary, Shabnam Vazifekhah, Mohammad Naghavi-Behzad, Mohammad Mirza-Aghazadeh-Attari

**Affiliations:** 1 Reproductive Health Research Center, Department of Obstetrics and Gynecology, Faculty of Medicine, Urmia University of Medical Sciences, Urmia, Iran; 2 Students’ Research Committee, Tabriz University of Medical Sciences, Tabriz, Iran; 3 Medical Philosophy and History Research Center, Tabriz University of Medical Sciences, Tabriz, Iran

**Keywords:** Premature Birth, Hypothyroidism, Thyroid Peroxidase, Premature Rupture

## Abstract

**Objective:** Preterm delivery is a common and eventful phenomenon with long standing complications, heavily burdening the health system. Many risk factors have been suggested to increase the likelihood of this event, one being hypothyroidism and high levels of anti-thyroid antibodies. The present study sought to explore the association between hypothyroidism and anti-thyroid antibodies with preterm delivery.

**Materials and methods:** A case control study was conducted on 400 patients attending Educational-Medical centers of Urmia University of Medical Sciences (Urmia, Iran) between November 2013 and April 2016, in which 200 patients with term deliveries and 200 patients with preterm deliveries were compared for differences in hypothyroidism, existence of anti- thyroperoxidase (TPO) antibodies based on blood samples obtained from the patients which were tested using chemi-luminescence method.

**Results:** In the group of patients with preterm delivery, 85 patients had hypothyroidism (42.5%), and from the term delivery group, 67 patients (33.5%) had hypothyroidism, the difference was not statistically significant (p = 0.14). But, when groups of early and late preterm deliveries were compared in terms of having anti-TPO antibodies, there was a significant difference between them, with early preterm delivery having 8 patients positive out of 44 patients and late preterm delivery having 7 positives out of 141 patients (p = 0.004).

**Conclusion:** Hypothyroidism had an insignificant effect on preterm delivery rates, but the existence of anti–TPO antibodies in the serum had a significant increasing effect on early preterm deliveries and could be regarded as a risk factor.

## Introduction

Delivery under 37 completed weeks is regarded as a preterm delivery which is subdivided to early and late preterm delivery (24-33 weeks and 34-36 weeks respectively) and it causes two-thirds of neonatal deaths (1, 2), also preterm delivery causes many complications, among which, some are earlier complications, such as respiratory distress syndrome, admission to neonate intensive care unit (NICU), intraventricular hemorrhage (IVH), and necrotizing enterocolitis (1, 3, 4). There are also long term complications in adulthood too, such as cardiovascular diseases, hypertension, kidney disease, diabetes, and neurodevelopmental disorders (5-7). Premature deliveries also are a major burden for the global health system financially, costing 18 billion dollars for the system (1), only regarding direct costs. There are many risk factors for preterm delivery, some being of crucial importance: history of preterm delivery, periodontal disease, low maternal body mass index (BMI), smoking, high maternal age, oligohydramnios, polyhydramnios, uterine anomalies, and maternal diseases (8, 9). Although many risk factors have been introduced, still more than half of the cases have no definite etiology (10). Normal thyroid function is necessary for pregnancy and fetal development (11, 12), so it would be expectable that there should be a relation between thyroid disorders and pregnancy complications (13). There was also an association between hypothyroidism and preterm delivery in some studies, for instance, Korevaar et al. demonstrated a 2.5-fold increase in the risk of preterm delivery by maternal hypothyroxinemia (14).

Noteworthy, It has been demonstrated that treatment with levothyroxine in asymptomatic pregnant women who had Anti-TPO antibodies, reduced the risk of abortion and preterm delivery (15). In a major study in Iran the prevalence of hypothyroidism was estimated to be about 13.7% (2.4% clinical and 11.3% subclinical). In this study a significant association between hypothyroidism and preterm delivery was shown (16). Study by Saki et al. showed that hypothyroidism increased the rate of fetal growth restrictions in 2.2-fold (16). In early pregnancy, consequent to high levels of beta-human chorionic gonadotropin (B-HCG), Thyroxine-binding globulin (TBG) and Total T4 increases in maternal serum, also maternal serum free T4 increases less significantly in parallel to raised B-HCG. It reaches a peak in the 12^th^ week, then it decreases to the normal level further, in parallel to decrease of B-HCG, serum thyroid-stimulating hormone (TSH) increases to its normal level after the early weeks’ suppression, noteworthy. There is no change in serum level of Anti-TPO antibody during this time period (1).

Although previously an association between presence of anti-TPO antibodies and preterm delivery was shown, evidence is not enough to consider that as a risk factor for preterm delivery or not (17, 18). Therefore, this study aims to further investigate the association of anti-TPO antibodies and hypothyroidism with mothers’ preterm delivery.

## Materials and methods

During the present case control study, which was conducted between November 2013 and April 2016 in Motahari Educational-Medical center of Urmia University of Medical Sciences (Urmia, Iran) a total of 400 patients were included in the study, in two groups of term and preterm delivery (each group having 200 patients) as the case and control groups. Two groups were matched for maternal BMI, number of fetuses, and background diseases such as diabetes and hypertension, drug history was studied in both groups for drugs potentially distorting thyroid function tests (19). All patients were fallowed up to 6 months after labor, and their TSH levels and existence of anti-TPO antibodies were tested to study any changes in the test results. No patient loss happened during the course of the study. Patients were clearly informed about the stages of the study and all patients whom were included in the study signed written informed consent forms and the study protocol was approved by the Regional Ethics Committee of Urmia University of Medical Sciences (Ethics code: Ir.umsu.rec.1394.62), which was in compliance with Helsinki Declaration.

Study sampling was conducted among patients with term and preterm delivery. In view of level of standard error of 7%, power of 80% and comparing the ratio of hypothyroidism based on TSH≥3mIU/l in case and control groups 0.1429 and 0.0569 respectively, 152 patients for each group was calculated. After all, 200 patients in each group (a sample of 400 patients with equal proportions in both groups) were collected and analyzed (noteworthy, the frequency of hypothyroidism was not 50 percent among the patients and hypothyroidism was less frequent but equal number of term and preterm cases were included).

Case group was composed of patients with preterm delivery (< 37 weeks), while in the conrol group there were only term deliveries. Inclusion criteria were consisted of singleton pregnancy, patients aging more than 18 years with no history of preterm delivery or thyroid diseases, where exclusion criteria included: uterine anomaly, age < 18 years, multiple gestation, previous preterm delivery, previous thyroid diseases, having diabetes and hypertension, and BMI < 18 or BMI > 30.

In the present study, 2cc of blood was obtained from the patients during the 8-12 weeks of pregnancy, and then the sample was preserved at -70 degrees of Celsius in the laboratory of the aforementioned center.

**Table 1:** Results of thyroid tests in patients with term, preterm and very preterm deliveries

		**Term Delivery** **n(%)**	**Preterm Delivery** **n(%)**	**Very Preterm Delivery** **n(%)**	**p**
Hypothyroidism	Normal	133 (66.5)	83 (56.1)	32 (61.5)	0.14
	+	67 (33.5)	65 (43.9)	20 (38.5)	
Anti-TPO antibodies	Normal	194 (97)	141 (95.3)	44 (84.6)	0.004
	+	6 (3)	7 (4.7)	8 (15.4)	

To evaluate hypothyroidism, TSH was investigated with chemi-luminescence method (LIAISON® XL, Diasorin, Italy). Amounts of TSH > 3mIU/L was documented as hypothyroidism, and anti-TPO antibodies > 90 IU/ml were considered positive (1, 20).

TSH and anti-TPO antibody levels in two groups with term and preterm delivery were compared. Statistical analysis was performed by Statistical Package for the Social Sciences (SPSS) software package version 16.0 for windows (SPSS Inc., Chicago, USA). Quantitative data were presented as mean ± standard deviation (SD), while qualitative data were demonstrated as frequency and percent (%). For statistical analysis, after determining distribution of continuous variables by Klomogrov Simirnov test, Independent sample t-test was applied to compare two group's results. Also collected data were studied using descriptive statistical methods, the mean difference test for independent groups, Chi Square^2 ^test or Fisher’s exact test. P value less than 0.05 was statistically considered significant in all steps. For calculating the odds ratio of increasing preterm delivery, in association to the existence of hypothyroidism and positive anti-TPO antibodies, single variable linear regression was used.

## Results

In the present study 400 women were studied, 200 having term (mean age: 28.07 ± 5.77) and 200 having preterm delivery (mean age 28.78±4.73), and the mean age difference between the two groups was not statistically significant (p = 0.50).

Hypothyroidism was seen in 67 patients (33.5%) in the term delivery group and in 85 patients (42.5%) in the preterm delivery group, difference between the two groups was not statistically significant (p = 0.14). Positive anti-TPO was reported in 15 patients (7.5%) from the preterm delivery group, and in 6 patients (3%) from the term delivery group, there was not a significant difference between the two groups in this category (p = 0.07). Results are summarized in table 1.

However, when groups of early and late preterm deliveries were compared in terms of having anti-TPO antibodies, there was a statistically significant difference between them, with early preterm delivery having 8 patients positive out of 52 patients and late preterm delivery having 7 positives out of 148 patients (p = 0.004). In patients belonging to the premature delivery group, 113 patients did not have premature rapture of membranes, from which 6 (5.3%) patients had positive anti-TPO antibodies, and 78 had premature rapture of membranes, from which 9 (10.3%) had positive anti-TPO antibodies. Results are summarized in table 2. The difference was not statistically significant (p = 0.18).

According to logistic regression, hypothyroidism increased the rate of preterm delivery 1.46 times, and presence of anti-TPO raised it 2.62 times, However, the difference wasn’t statistically significant (p = 0.06 and p = 0.05 respectively).

In the fallow up conducted 6 months after labor, no change in the thyroid function tests were observed (69 patients with hypothyroidism in the preterm delivery group and 84 patients with hypothyroidism in the term delivery group (p = 0.16), 17 patients with anti-TPO antibodies in the preterm delivery group and 7 patients with negative anti-TPO antibodies in the term delivery group (p = 0.08), 9 patients in the early premature delivery vs. 8 in the late premature delivery (p = 0.005) and finally 7 patients had anti-TPO antibodies in the group without premature rapture of membranes compared to 10 patients with anti-TPO positive in the group with premature rapture (p = 0.19).

**Table 2:** Results of thyroid tests compared to the time of rapture of the membranes

		**Premature Rapture** **n(%)**	**Normal** **n(%)**	**p**
Hypothyroidism	Normal	53 (60.9)	62 (54.9)	0.39
	+	34 (39.1)	51 (45.1)	
Anti-TPO antibodies	Normal	78 (89.7)	107 (94.7)	0.18
	+	9 (5.3)	6 (10.3)	

## Discussion

Normal thyroid function is essential during pregnancy and normal amounts of thyroid hormones are responsible for adequate fetal growth. One reason of thyroid hormone deficiency is auto-immunity, induced by anti-TPO antibodies. The exact effect of these antibodies on pregnancy is not known but it has been believed that they are related with low reserves of thyroid hormones (21), this relation is further apparent in very premature labor (22).

In the present study, the association of preterm delivery with hypothyroidism and anti-TPO antibodies was evaluated. The results were as 33.5% Hypothyroidism in the term, and 42.5% in the preterm delivery group. Although hypothyroidism was higher in the preterm delivery group, the difference was not statistically significant. 

Green et al. conducted a similar study, finding that hypothyroidism, and TSH > 3mIU/L almost tripled the chance of very preterm delivery (< 32W). However similar to the current study, results were not significant. But contrarily to the present study a significant association was not found between anti TPO antibodies and early premature deliveries (14). 

In a study done by Korevaar et al., there was a significant association between preterm delivery and hypothyroidism, the latter being associated with a 2.5-fold increased preterm delivery, 3.6-fold increase in very preterm delivery, which did not follow the present study, although the difference might be caused by the higher population undergoing the study in the aforementioned one (5971 versus the 400 people included in the present study). Also In this study done by Korevvar et al. anti-TPO antibodies increased the risk of preterm delivery in 1.7-fold (p = 0.01) and the risk of very preterm delivery 2.5-fold (p = 0.02), which was in concordance to the present study, but it is worth to note that the latter study suggests that even by correcting the thyroid stimulating hormone level and T4, the risk does not decline, showing that anti-TPO antibodies could be seen as an independent risk factor for preterm delivery (14).

Tierney et al. (23) didn’t find any association between hypothyroidism and high levels of anti-TPO antibodies and preterm delivery, similar to the p resent study.

In a systematic review consisted of 8 studies and more than 39000 patients (20), there was a significant association between clinical (OR = 1.25, 95% CI: 1.15-1.36, p < 0.01) and subclinical (OR = 1.25, 95% CI: 1.14-1.36, p < 0.01) hypothyroidism, the results of this study did not correlate with the results of the present study.

In our study, anti-TPO antibodies were positive in 7.5% of preterm deliveries, and in 3% of term deliveries. However, it wasn’t statistically significant. When we compared two groups of late and early preterm delivery (< 32 weeks), anti-TPO was more common in very preterm group and it was statistically significant.

He et al. (24) found that, there was a significant association between preterm delivery and elevated anti-TPO antibodies. In their study, elevated anti-TPO increased the risk of preterm delivery in 1.69-fold (p = 0.003). Similarly, Haddow et al. (17) showed that high levels of anti-TPO antibodies increased the risk of preterm delivery.

But Haddow et al. (17) showed that preterm delivery with ruptured membranes was more common in mothers with elevated anti- TPO.

Similarly, Soto-Rivera et al found that preterm delivery with ruptured membranes was more common in pregnant women with elevated anti-thyroid Antibodies. but in contrast to the present study, a significant association was not seen between early premature delivery and anti–TPO levels, although the study was in concordance to the present study ’s result, in not having a significant association between anti-TPO antibodies and preterm delivery (early and late premature delivery) (18).

Recently Vissenberg et al. in a study at 2015 (25), suggested that treating women with anti-TPO antibodies with levothyroxine could have a beneficial effect. It is also important to highlight that in many auto immune diseases anti-TPO antibodies are elevated. Keeping this in mind, a regimen of similar drugs could be suggested for women in danger of such diseases with high anti-thyroid antibodies, in order to reduce the likely-hood of preterm delivery.

The suggestions of the present study could be the point that same comparisons should be done on higher number of patients, and also new studies should consider the effect of diabetes, smoking, and other possibly corrosive factors not included in the present study. Finally, studying the association between hypothyroidism and pre-eclampsia could be of merit.

***Limitations:*** One limitation of the study was the numbers of the centers in which the study was conducted was limited, and for further generalizing the results there would be need for more centers to be included.

## Conclusion

The present study showed a significant association between anti-thyroid antibodies and early preterm delivery, and unlike previous studies, it did not show a significant association between preterm delivery (early and late combined) and hypothyroidism or preterm delivery and anti-TPO antibodies. Also no significant association was found between anti-TPO levels in preterm deliveries and premature rapture of membranes.
